# The Effect of Implicit–Explicit Followership Congruence on Benevolent Leadership: Evidence from Chinese Family Firms

**DOI:** 10.3389/fpsyg.2016.00812

**Published:** 2016-06-07

**Authors:** Xiao Wang, Jian Peng

**Affiliations:** School of Management, Jinan UniversityGuangzhou, China

**Keywords:** positive followership prototype, positive followership trait, benevolent leadership, implicit followership theory, polynomial regression

## Abstract

Benevolent leadership, a traditional Chinese leadership style generated under the influence of Confucianism, has been under growing discussion since its proposal. However, existing research has focused mainly on the consequences of benevolent leadership, and research probing into its antecedents is scarce. To fill such research gap, the current study aims to explore the effect of the congruence between implicit positive followership prototype (PFP) and explicit positive followership trait (PFT) on benevolent leadership. Polynomial regression combined with the response surface methodology was used to test the hypotheses herein. The results, based on a sample of 241 leader–follower dyads from four Chinese family firms, indicated the following: (1) benevolent leadership is higher when leader PFP is congruent with follower PFT than when they are incongruent; (2) in cases of congruence, benevolent leadership is higher when leader PFP and follower PFT are both high rather than low; (3) in the case of incongruence, there is no significant difference for the level of benevolent leadership in two scenarios: “low leader PFP – high follower PFT” and “high leader PFP – low follower PFT”.

## Introduction

Traditional Chinese Confucianism has advocated harmonious interpersonal relations since ancient times; these relations are manifested in the context of power relations between a benevolent monarch and his loyal subject or a kind father and obedient children (Cheng et al., 2004). As expressed in this type of harmonious ideology, a traditional style of leadership — benevolent leadership — was generated in Chinese social organization (Cheng et al., 2000). Benevolent leadership refers to leaders treating followers as family members, showing concern for followers’ well-being in both the work domain and private life (Wang and Cheng, 2010). Compared to the individualized consideration dimension of transformational leadership that limits its consideration for followers within the work domain (Bass, 1985), benevolent leadership expresses that consideration extends from the work domain to the non-work domain, such as assisting followers during their personal crises and showing individualized concern beyond professional relationships (Cheng et al., 2000).

To date, some studies provide evidence that benevolent leadership not only can affect followers’ job attitudes, but also can boost followers’ job performance and creativity (Wang and Cheng, 2010; Chan and Mak, 2012). Unfortunately, the majority of researchers mainly focus on the consequences of benevolent leadership, whereas the research probing into its antecedents proves rare, which directly causes the following question “why does Chinese leadership remain benevolent?” to remain generally unanswered. To clarify this issue, the current study aims to explore the antecedents of benevolent leadership.

In recent years, the application of social cognitive theory to the research field of followership expedites the implicit followership theory (Sy, 2010), which makes it possible to uncover the antecedents of benevolent leadership. The implicit followership theory proposes that individuals generate personal assumptions about the traits that characterize followers (Sy, 2010). These assumptions are stored in the mind as followership prototypes and are activated when individuals interact with actual followers. Based on the valence of a prototype, Sy (2010) classified followership prototypes into the positive followership prototype (PFP) and the negative followership prototype. Following the positive psychology movement, PFP is gradually becoming a main research focus. PFP comprises the assumed traits characterizing good followers, such as industriousness, enthusiasm and good citizenship (Sy, 2010). Existing research finds that leader PFP can not only improve the performance expectations for followers and transformational leadership (Duong, 2011; Whiteley et al., 2012) but also effectively improve followers’ job satisfaction and job performance (Duong, 2011). In compliance with the research approach for positive psychology, this paper focus on the theme of PFP.

According to the implicit followership theory, in organizational settings, leaders’ PFP will be activated unconsciously and compared with the followers’ explicit/actual followership traits (Epitropaki et al., 2013) in the leader–follower interaction. The leader acts in accordance with the outcome of these comparisons (Shondrick and Lord, 2010). Explicit/actual followership traits consist of two valences: positive and negative in which a positive followership trait (PFT) is regarded as welcome and excellent; such traits manifest in excellent work ability, positive affect and gracious morality. Based on recognition-based cognitive process (Epitropaki et al., 2013), the present study proposes that leaders will form an impression of their followers in light of the congruent degree between leader PFP and follower PFT, and it is a determining factor for leaders to treat followers with some level of benevolent leadership.

According to the levels of leader PFP and follower PFT, we have identified the following four different matching scenarios, as shown in **Table 1**: high–high, low–low, high–low, low–high. The former two fall into the category of congruence, and the latter two fall into the category of incongruence. Exploring the effect of implicit–explicit followership congruence on benevolent leadership, we will address the following: (1) whether benevolent leadership is higher in congruence scenarios than in incongruence scenarios, (2) whether benevolent leadership is higher in a high–high scenario than in a low–low scenario between the two congruence scenarios, and (3) whether benevolent leadership is higher when leader PFP is at a lower level than follower PFT between the two incongruence scenarios in comparison to the opposite scenario.

**Table 1:** The four different scenarios of (in) congruence between leader PFP and follower PFT.

		Leader PFP	
		Low	High
**Follower PFT**	**Low**	Congruence scenario Low leader PFP-Low follower PFT	Incongruence scenario High leader PFP-Low follower PFT
		
	**High**	Incongruence scenario Low leader PFP-High follower PFT	Congruence scenario High leader PFP-High follower PFT

This study contributes to the literature in several aspects. First, we contribute to the work on benevolent leadership by exploring its antecedents from the perspective of leader–follower congruence. We particularly focus on PFP–PFT congruence and its relationship with benevolent leadership. The congruence perspective can provide us with an access to a complete understanding of why some leaders treat their followers benevolently. Intriguingly, prior research has found that leaders have a tendency to evaluate followers who are professionally similar to them positively (e.g., Gioia and Sims, 1985). As Giorgi et al.’s (2014) summarized, mentors often select protégés who share the similar characteristics with them. Some promotions and good performance appraisals seem to be influenced by this evaluation bias. Besides, Giorgi et al. (2014) have found that incongruence (leader–follower) in stress contexts was negatively related to followers’ psychological well-being and workplace bullying played a mediator role in such relationship. Drawing on the arguments above, we expect that the congruence between leader PFP and follower PFT may elicit leaders’ benevolent leadership behavior, and any incongruence may trigger negative emotions and relationship conflicts eventually hindering the emergence of benevolent leadership. Second, we contribute to the work on asian models of leadership by introducing the construct of benevolent leadership. Asian economy are growing fast and have doubled in size to almost 45 trillion dollars since 1980 (Arvey et al., 2015). Such rapid economic growth indicates rapid growth in the number of leadership positions. However, disappointingly, the growth of scholar research on leadership in Asia has not been as fast as its economy. On account of that situation, many calls have been made for an examination of leadership in asian contexts. This study investigates the traditional Chinese leadership style, namely benevolent leadership, attempting to respond to such calls.

## Theoretical Background and Hypotheses Development

### Implicit Followership Theory

Implicit followership theory posits a recognition-based cognitive process in which leaders are inclined to evaluate followers on the basis of the perceived congruence between their actual followership traits and the attributes of a pre-existing followership prototype (Epitropaki et al., 2013). More specifically, leaders are likely to form a positive impression of the followers in the case of congruence and thus give more resources and support to the followers (Coyle and Foti, 2015). Otherwise, the leaders will form a negative impression of the followers. Drawing on the arguments mentioned above, we infer that benevolent leadership is positively related to the congruence of leader PFP and follower PFT.

### Implicit–Explicit Followership (in) Congruence and Benevolent Leadership

Leaders who possess PFP will form positive expectations for followers (Whiteley et al., 2012), whereas followers possessing PFT appear to possess excellent work ability, positive affect, and gracious morality. When congruence between leader PFP and follower PFT occurs, the followers’ actual followership traits can satisfy the positive expectations of leaders. On one hand, the satisfaction of expectations will improve the leaders’ trust in the followers, which makes leaders more willing to assign other roles to the followers (Dienesch and Liden, 1986), as well as provide them help to fulfill the assigned role. On the other hand, it will increase the leaders’ favor of followers and facilitate the development of socio-emotional relationships between them (Coyle and Foti, 2015). Under such conditions, followers are more likely to become in-group members in leader–follower interaction. In Chinese organizations, Cheng (1995) proposed that paternalistic leaders would be considerate towards the in-group members in both work and non-work domains, demonstrating a high level of benevolent leadership.

In the case of incongruence between leader PFP and follower PFT, followers can hardly meet the leaders’ expectations, which will cause the dissatisfaction of leaders and destroy the trust and exchange relationship between them (Bashshur et al., 2011). Furthermore, this will lower the possibility of the leaders’ consideration for the followers’ well-being. For example, when the leader PFP is higher than the follower PFT, leaders have high expectations for the followers. However, the actual followership traits are inferior to the leader’s expectations, which may lead to the leaders’ disappointment and reduction of consideration for the followers. In the situation of the leader PFP being lower than follower PFT, followers’ actual followership traits are beyond leaders’ expectations, which may induce feelings of uncertainty in leaders. Unfortunately, the sense of uncertainty will breed egoism, thus leaders will consider others’ interests less (Todd et al., 2015). Furthermore, leaders with a sense of uncertainty will decrease their consideration for their followers, and be even skeptical of the motivations of the followers (Frese and Fay, 2001). Under such circumstances, benevolent leadership is lower than the congruence situation. Therefore, we present the following hypothesis:

#### Hypothesis 1

Benevolent leadership is higher when leader PFP is congruent with the follower PFT than in the opposite situation.

### Benevolent Leadership in the Case of Congruence

While discussing congruence, there are two congruence scenarios: high leader PFP-high follower PFT (high–high) and low leader PFP-low follower PFT (low–low). we assume that a benevolent leadership should emerge with the rising of PFP and PFT within congruence scenarios. This is because benevolent leadership in the high–high congruence scenario can not only benefit from congruence but also from high PFP and PFT. Based on the sense-making function of PFP (Sy, 2010), leaders with high PFP are likely to act more benevolently (McGregor, 1960) when followers’ high level of PFT can meet the high expectations of leaders. Moreover, followers with high PFT may receive more support and consideration from their leaders as a social exchange (Blau, 1964) process.

In contrast, in the low–low congruence scenario, although leaders and their followers have the benefits of implicit–explicit followership congruence, the benefit may be undermined due to the reduced level of PFP and PFT. In such a condition, although the followers’ actual followership can still meet leaders’ expectations, the gap between the efforts made to meet low expectations and those made to meet high expectations makes leaders’ concerns for followers in a low-low congruence scenario remain weak. Taking these two aspects into consideration, benevolent leadership becomes stronger than in a high–high congruence situation.

#### Hypothesis 2

Benevolent leadership is higher when leader PFP and follower PFT are both high rather than low.

### Benevolent Leadership in the Case of Incongruence

In the case of incongruence, we infer that there exists difference in benevolent leadership between the two scenarios: high leader PFP-low follower PFT and low leader PFP-low follower PFT. When leader PFP is lower than follower PFT, followers’ actual followership are beyond leaders’ expectations. Despite the fact that leaders have a sense of uncertainty and a low positive impression of followers, leaders may provide some basic material or psychological resource for followers based on the principle of reciprocity (Blau, 1964), by which the negative influence of incongruence on benevolent leadership will be alleviated. While leader PFP is higher than follower PFT, leaders pin higher hopes on the followers (Whiteley et al., 2012), but the followers’ actual followership can hardly agree with the presupposition of leaders. Thus, leaders would show disappointment and dissatisfaction toward the followers, which affect their attitude and behavior toward the followers in the resulting process (Festinger, 1962), such as in the reduction of the level of concern and offering of resources in both work and personal life domains. Furthermore, leaders may even adopt some negative leadership styles to treat followers (Mawritz et al., 2014). In conclusion, the current paper hypothesizes that:

#### Hypothesis 3

Benevolent leadership is higher when leader PFP is at a lower level than follower PFT in comparison to the opposite scenario.

## Materials and Methods

### Participants

We took the employees varied in demographic characteristics from four Chinese family firms in food industry as the participants in our study. Each of these firms is a commercial organization where decision-making is affected by multiple generations of a family who are closely identified with the firm through ownership or leadership. The firms’ size range from 131 to 342 with an average size of 229.71.

In our study, 274 leader–follower dyads agreed to fill out surveys. In actually, 253 leader–follower dyads filled in the questionnaire, reaching a 92.34% response rate. After some questionnaire copies were excluded because they had too many unanswered questions or had identical answers, the final sample consisted of 241 leader–follower dyads. Among the final sample, leaders were primarily men (69.76%) with an average age of 35.62 (*SD* = 5.60), and they had 16.91 years (*SD* = 3.43) of education on average. The majority of followers (54.36%) were women with an average age of 28.95 (*SD* = 6.87). They had 18.89 years (*SD* = 4.93) of education on average and had been working with their leader for an average of 2.98 years (*SD* = 7.20).

### Procedure

We first got in touch with the human resource director of each firm and then asked whether their firms are willing to participate in this survey. After getting approval from their firms, the director of each company introduced an inside helper for recruiting teams of participants from their own companies for this survey. Most of the helpers are human resource department staff who are equipped with job experience in personnel assessment. When the inside helpers for each company were selected, we briefed them on the purpose of this study, proper ways of collecting data in addition to precautions to notice in the survey. One thing worth to mention is that the first author of this paper also took part in the distribution and collection of the questionnaires as he offered guidance and assistance for the inside helper.

The research has been performed in accordance with the recommendations of the Science & Technology Research Office of JNU. There were no unethical behaviors in the research process, and we were exempt from further ethics board approval since our study did not involve human clinical trails or animal experiments. The form of questionnaires were paper–pencil testing. In the first page of our questionnaire, consent was presented and participants were informed that they were completely free to join or drop out the survey. Only those who were willing to participate were recruited.

According to who agreed, we summarized a name list and assigned a number to each participant so that we can pair followers’ questionnaire with leaders’. To carry out the survey, we first gave out questionnaires to followers and then to leaders, assuring them that the results of the survey would be kept confidential completely, and will be used only for academic research. To fulfill their roles in the survey, followers reported their perceptions of benevolent leadership and PFT, and leaders reported their PFP. To show our appreciation, a high-quality pen was awarded to each participant who completed the questionnaire.

### Measures

The questionnaires of PFP and PFT used in the present study were originally in English. However, the participants surveyed in the study were leaders and followers from Chinese family firms. Thus, we needed to translate the English scales into Chinese so as to make them understood by the participants. In the process, we took translation/back translation procedures (Brislin, 1986)^1^ because the method had been widely used in studies in this regard in non-English speaking countries (Chen et al., 2016).

Prior research has expressed concern about possible cultural difference in response style regarding the use of rating scales. For example, Chen et al. (2006) found that Chinese were more likely than Americans to use the midpoint on the scales. This phenomenon may be explained by the virtues of moderation promoted by Confucian philosophy in China which make Chinese people believe that they should not stand out from the group. In order to reduce the participants’ response biases in favor of midpoint, a six-point Likert-type scale was used for all the measures instead of widely used five-point or seven-point Likert-type scale.

#### Positive followership prototype

We assessed PFP with a 9-item measure consisting of three sub-dimensions with three items each, including Industriousness (productive, hardworking, goes above, and beyond), Good Citizenship (reliable, loyal, and team player) and Enthusiasm (outgoing, excited, and happy) (Sy, 2010). According to Sy (2010) and Whiteley et al. (2012)’s suggestion, leaders were asked to rate how characteristic each of the nine items was of a follower, with no definition of the term provided. (α = 0.88). To examine convergent validity, we conducted confirmatory factor analysis in which the three items of each sub-dimension were modeled as indicators of their respective latent constructs (Cunningham et al., 2001). The average variance extracted (AVE) values for the three latent factors (0.67, 0.50, 0.71) are above 0.50, which provides evidence for the convergent validity of PFP measurement.

#### Positive followership trait

According to Epitropaki and Martin (2005)’s research, PFT was assessed using the aforementioned 9-item scale based on the traits identified by Sy (2010), but the instructions of PFT were changed. Followers were asked to rate how the characteristics applied to themselves. (α = 0.95). We also conducted confirmatory factor analysis to examine the convergent validity of PFT measurement. The results showed that the AVE values for the three latent factors (0.71, 0.72, 0.56) are above 0.50.

#### Benevolent leadership

We measure benevolent leadership using an 11-item scale developed by Cheng et al. (2000). The scale is constructed by two sub-dimensions, namely individualized care within work domain and non-work domain. Sample items include “My superior encourages me when I’m faced with job dilemmas,” and “Beyond work relations, my superior shows concern about my daily life.” (α = 0.91). The full list of items in English version can been see from Cheng et al. (2004). The AVE values for the two latent factors (0.64, 0.49) are above or close to 0.50.

#### Control variables

To be consistent with the previous research on person-supervision fit theory (Zhang et al., 2012; Matta et al., 2015), we controlled for the similarity between leaders and followers in gender, age and years of education, which was operationalized by the absolute difference score between leader and follower. According to prior research on leadership in Asian settings (Giorgi et al., 2013), we also controlled for firm size and dyadic tenure. Dyadic tenure referred to the period of time for which a follower worked with his or her leader.

### Data Analysis

We analyzed the data in several steps. First, we carried out descriptive statistics and correlation analysis by using SPSS 19.0, and then we tested measurement invariance and discriminant validity using Amos 17.0. Next, we examined the congruence/incongruence effect by the means of polynomial regression combined with the response surface methodology (Edwards and Parry, 1993).

In polynomial regression, benevolent leadership was regressed on control variables as well as the five polynomial terms, that is, leader PFP, follower PFT, leader PFP squared, follower PFT squared and the interaction between leader PFP and follower PFT. In other words, we estimated the following equation (to make it as simple as possible, we omitted all control variables)

(1) $$Y=b_{0}+b_{1}L+b_{2}F+b_{3}L^{2}+b_{4}(LF)+b_{5}F^{2}+e\,$$

where *Y* stands for the dependent variable (i.e., benevolent leadership), and *L* and *F* for leader PFP and follower PFT, respectively. To reduce multicollinearity and facilitate interpretation of the results, we mean-centered *F* and *L* before generating the square and interaction terms. Next, in accordance with the regression coefficients estimated by the equation, we plotted the three-dimensional response surface in which *F* and *L* were plotted on the perpendicular horizontal axes, and *Y* was plotted on the vertical axis (Edwards and Parry, 1993).

Hypothesis 1 predicted a congruence effect, which can be tested based on the following features: first, the curvature along the incongruence line (*b*_3_–*b*_4_+*b*_5_) had to be significant and negative, that is to say, the surface along the incongruence line should be an inverted U-shape one; Second, the intercept and slope of the first principal axis of the response surface should not be significantly different from 0 and 1, respectively, so as to indicate that the ridge of the response surface (i.e., the first principal axis) is located along the congruence line (*L* = *E*), ensuring that benevolent leadership is maximized when the leader PFP is congruent with follower PFT (Edwards and Cable, 2009).

Hypothesis 2 explored the cases where leader PFP and follower PFT were congruent at either high or low level. We tested it by examining the slope of the congruence line (*b*_1_–*b*_2_). This feature determines whether the surface along the congruence line is flat or varied. A significant and positive slope could support Hypothesis 2.

Hypothesis 3 predicted an incongruence effect, which can be tested on the slope of the incongruence line (*b*_1_–*b*_2_), a significant and negative slope could support Hypothesis 3.

## Results

### Correlations among Study Variables

**Table 2** shows the means, standard deviations, and intercorrelations of the variables. The results indicate that both leader PFP (*r* = 0.15, *p* < 0.05) and follower PFT (*r* = 0.21, *p* < 0.001) are positively correlated with benevolent leadership.

**Table 2:** Means, standard deviations, and correlations.

	*M*	*SD*	1	2	3	4	5	6	7
1. Gender similarity	0.42	0.49							
2. Age similarity	8.15	5.27	-0.05						
3. Education similarity	5.56	3.51	-0.03	0.01					
4. Dyadic tenure	2.97	7.20	0.13	-0.39^∗∗∗^	-0.02				
5. Firm size	229.71	79.67	0.13^∗^	-0.08	0.03	0.12			
6. Leader PFP	4.75	0.93	-0.13	0.20^∗∗^	0.06	0.02	0.01		
7. Follower PFT	3.98	1.02	0.18^∗∗^	-0.37^∗∗∗^	-0.00	0.30^∗∗∗^	0.61^∗∗∗^	0.05	
8. Benevolent leadership	4.07	0.74	0.08	-0.05	0.02	0.04	0.04	0.15^∗^	0.21^∗∗∗^

*N = 241; ^∗^p < 0.05; ^∗∗^p < 0.01; ^∗∗∗^p < 0.001.*

### Confirmatory Factor Analyses and Discriminant Validity

We conducted confirmatory factor analyses to examine the discriminant validity of the variables, namely leader PFP, follower PFT and benevolent leadership. It can be seen from **Table 3** that the chi-square of either of the other models (M2–M5) shows a significant increase compared to that of the three-factor model(M1), and the three-factor model(M1) is obviously better in the other fit indices (Hu and Bentler, 1999), so we concluded that the three variables were empirically distinct from each other, representing three distinct constructs.

**Table 3:** Confirmatory factor analyses.

Model	*χ*^2^	*df*	Δ*χ*^2^ (Δ*df*)	RMSEA	RMR	CFI	GFI	NFI
M1: PFP; PFT; BL	48.34	17	—	0.08	0.07	0.97	0.95	0.96
M2: PFP+PFT; BL	256.27	19	207.93 (2)	0.23	0.21	0.79	0.78	0.78
M3: PFP; PFT+BL	209.58	19	161.24 (2)	0.20	0.11	0.83	0.85	0.82
M4: PFP+BL; PFT	218.61	19	170.27 (2)	0.21	0.12	0.83	0.84	0.82
M5: PFP+PFT+BL	409.70	20	361.36 (3)	0.29	0.22	0.66	0.72	0.65

*N = 241. BL, benevolent leadership. All alternative models are compared to the hypothesized model (M1). All Δχ^2^ are significant at p < 0.001.*

### Hypothesis Testing

**FIGURE 1:**
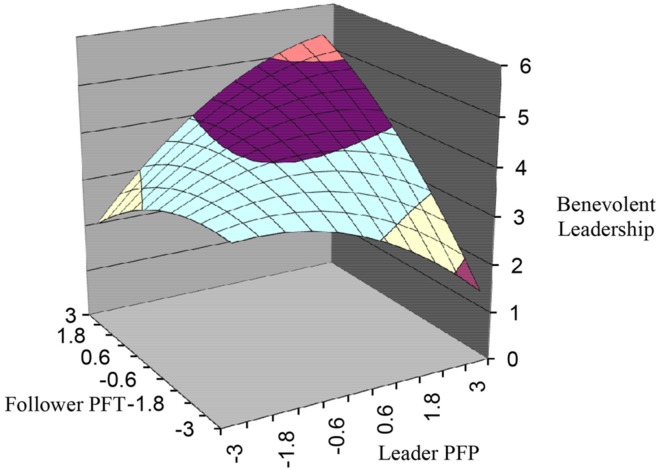
**Response surface analysis**.

Hypothesis 1 predicts that benevolent leadership is higher when leader PFP is congruent with the follower PFT than when they are incongruent. The results of the test of Hypothesis 1 are shown in **Table 4**: *a*_4_ is significant and negative (*a*_4_ = -0.26, *p* < 0.05), and also the 3 s-order polynomial terms are jointly significant in predicting benevolent leadership (*F* = 3.49, *p* < 0.001), and they explain significant incremental variance in benevolent leadership (Δ*R*^2^ = 0.05, *p* < 0.05). Shown in **Figure 1**, the response surface along the incongruence line is an inverted U-shape one, indicating that benevolent leadership is higher when leader PFP and follower PFT are congruent.

**Table 4:** Polynomial regressions.

Variable	Benevolent leadership		
	Model 1	Model 2	Model 3
Constant	4.00^∗∗∗^	4.21^∗∗∗^	4.37^∗∗∗^
Age similarity	0.10	0.10	0.12
Gender similarity	-0.01	-0.01	-0.00
Education similarity	0.01	0.00	0.01
Dyadic tenure	0.00	-0.02	-0.02
Firm size	0.00	0.00	-0.00
Leader PFP (b_1_)		0.12^∗^	0.13^∗^
Follower PFT (b_2_)		0.21^∗∗∗^	0.23^∗∗^
Leader PFP^2^ (b_3_)			-0.06
Leader PFP × Follower PFT (b_4_)			0.16^∗∗∗^
Follower PFT^2^ (b_5_)			-0.04
*F*	0.42	3.04^∗∗^	3.49^∗∗∗^
*R*^2^	0.01	0.08	0.13
Δ*R*^2^		0.07^∗∗∗^	0.05^∗^
*Congruence Line* (*L* = *F*)			
Slope *a*_1_ (*b*_1_+*b*_2_)	0.36^∗∗∗^		
Curvature *a*_2_ (*b*_3_+*b*_4_*+b*_5_)	0.06		
*Incongruence Line* (*L* = -*F*)			
Slope *a*_3_ (*b*_1_–*b*_2_)	-0.10		
Curvature *a*_4_ (*b*_3_-*b*_4_*+b*_5_)	-0.26^∗^		

*N = 241; ^∗^p < 0.05; ^∗∗^p < 0.01; ^∗∗∗^p < 0.001.*

In order to further support the congruence effect, we examined the slope and the intercept of the ridge of the response surface. The results show that the 95% bias-corrected “bootstrap” confidence intervals for the slope and the intercept are, respectively, (0.36, 3.15) and (-0.99, 1.21), indicating that the slope and the intercept of the first principal axis are not significantly different from 1 and 0, respectively. Thus, the ridge of the response surface is located along the congruence line, ensuring that benevolent leadership is maximized when PFP are congruent with PFT. In conclusion, Hypothesis 1 is verified.

Hypothesis 2 predicts that benevolent leadership is higher when leader PFP and follower PFT are both high instead of being both low. **Table 4** shows that a_1_ is significant and positive (a_1_ = 0.36, *p* < 0.001). After further checking the response surface (**Figure 1**), we could find that benevolent leadership at the rear corner (where F = L = 3) is higher than that at the front corner (where F = L = -3). Hypothesis 2 is therefore verified.

Hypothesis 3 predicts that benevolent leadership is higher when leader PFP is at a lower level than follower PFT in comparison to the opposite situation. **Table 4** shows that a_3_ isn’t significant (a_3_ = -0.10, *ns*). Hence, Hypothesis 3 is not verified.

## Discussion

Although existing research indicates that leader PFP plays an essential role in leadership behavior, there is still less knowledge about whether leader PFP and follower PFT combine to jointly influence benevolent leadership. To address this issue, the current paper investigates the effect of congruence of leader PFP and follower PFT on benevolent leadership using questionnaire surveys in the context of Chinese culture. The results show the following: (1) benevolent leadership is higher when leader PFP is congruent with follower PFT than in the case of incongruence; (2) benevolent leadership is higher when leader PFP and follower PFT are both high rather than low; (3) there is no significant difference for the level of benevolent leadership in two incongruence scenarios.

### General Discussion

First, this study attempted to capture the recognition-based cognitive process in which leaders are assumed to engage in so as to evaluate the followers and eventually determine an adequate response toward the followers. Specifically, we focused on the role of implicit–explicit followership congruence in benevolent leadership. Using polynomial regression analysis, we found evidence for the positive relationship between PFP–PFT congruence and benevolent leadership. The leaders were found to be benevolent in the interaction with their followers when the followers’ explicit PFT was perceived to be close to leaders’ implicit PFP. According to cognition category theory (Lord et al., 1984), leaders compare the follower’s explicit characteristics with their followership prototype and any discrepancies that derived from that comparison are assumed to shape the leader’s impression of the followers. The more a follower displays what the leader believe to be the characteristics of a good follower, the more favorably the leader respond to the follower (van Gils et al., 2010) and the more they are willing to treat the follower benevolently. Nevertheless, in the opposite case, the follower’s explicit characteristics are not in compliance with the assumed expectation of leaders, which may hinder benevolent leadership.

The leaders’ emotions (satisfaction with or liking for followers) aroused by implicit–explicit followership congruence also play a key role in shaping benevolent leadership behavior. Considering PFP serves as a sense-making function that act as an antecedent of leaders’ emotions toward followers (Sy, 2010), leaders may be satisfied with and liking for followers when followers’ PFT match their PFP. Such positive emotions have been associated with positive social interactions, such as prosocial behavior and individual support for followers (Rubin and Bommer, 2005). Hence, positive emotions induced by PFP–PFT congruence contribute to the emergence of benevolent leadership behavior. Prior research found that leader PFP could predict transformational leadership (Duong, 2011), overlooking the role follower PFT plays in this process. In fact, leaders possessing PFP do not necessarily show a high level of positive leadership on account of leaving out follower PFT.

We found that benevolent leadership will increase with the rising PFP and PFT under the congruence scenario. Lord and Maher (1993) suggest that cognitive prototype serves not only as a basis to interpret the characteristics of the dyad partner, but also as a foundation for own behavior. That is because individuals’ behavior is oriented by cognition to a great extent (Chen and Bargh, 1997). Leaders can thus be assumed to act in compliance with their PFP, which means that leaders who hold a PFP will resort to a more benevolent leadership (McGregor, 1960). Similarly, followers’ personal traits will also influence leadership (Lapierre and Bremner, 2010). When followers show more PFT, assistance and concern will be granted from the leaders as a reward. The discovery mentioned above not only gives empirical evidence to the XY theory (McGregor, 1960) but also further clarifies the positive relationship between benevolent leadership and PFP, PFT.

Finally, the difference in benevolent leadership was not statistically significant in the two incongruence scenarios. Recently, theorists have begun to realize that leadership is an interactive process in which both party of the leader–follower dyad play a vital role (Derue and Ashford, 2010). Hence, leaders and followers may contribute to the benevolent leadership equally based on their unique roles. This may explain why there was no significant differences in the level of benevolent leadership of two incongruence scenarios (low leader PFP – high follower PFT and high leader PFP – low follower PFT). According to the social exchange theory, a high quality leader–follower relationship characterized by mutual trust and support can be reached when both party benefit equally. However, the contributions and benefits in leader–follower interaction in the incongurence scenarios are not equal, despite PFP is higher than PFT or otherwise, which makes leaders tend to form a bad impression of the follower and will show little consideration for the followers.

### Theoretical Implication

This paper broke through the limitations of existing benevolent leadership research, which predominantly focuses on the consequences of benevolent leadership (Wang and Cheng, 2010; Chan and Mak, 2012); this study attempts to reverse the emphasis toward the antecedents of benevolent leadership. Giving evidence in support of the positive relationship between implicit–explicit followership congruence and benevolent leadership, the present study fills the aforementioned research gap and opens the black-box of the antecedents of benevolent leadership. Hence, we make theoretical contributions to the benevolent leadership literature.

In addition, this study provides new proof of person-supervision fit theory. Recently, some research indicated that positive effects such as job attitude and performance will be achieved because of leader–follower congruence in personality and cognition (Zhang et al., 2012; Carter and Mossholder, 2015). Coyle and Foti (2015) proposed that followership prototype congruence would facilitate corporation. Epitropaki and Martin (2005) demonstrated that the implicit–explicit leadership prototype congruence could improve leader-member exchange and followers’ job attitude. Consistent with the above-mentioned points, we also found that leader–follower congruence will boost positive impacts.

### Practical Implication

Benevolent leadership, a popular and efficient Chinese leadership style among followers, plays an essential role in the operation and development of organizations (Cheng et al., 2000). Despite that Confucianism molds the style of benevolent leadership, different leaders will perform at different levels of benevolent leadership, which also includes a low level of benevolent leadership. Therefore, how to develop benevolent leaders is an on-going topic in managerial practice.

Previous studies concentrate on developing leaders by means of leadership training activities, thus comparatively neglecting improving leadership via followership training. Research on positive psychology interventions offers some insights into how followership can be promoted to be more positive. For instance, organizations could facilitate these efforts by organizing team building activities. Such activities could increase followers’ reflection on what characteristics they should be and how they could be an effective follower, and positive norms for leader – follower interaction are created at the same time, all of which ultimately help followers learn the PFTs such as team player, good citizen and loyalty.

We also provide a refreshing view for the development of benevolent leadership, that is, that an organization should equip its followers with some positive traits in accordance with leaders’ positive expectations, which will provide a means for the leaders to generate a positive attitude toward their followers and show benevolent leadership. At the same time, leadership training is also just as important. In consideration of the guiding role leader PFP plays in leadership behavior, we propose that organizations should incorporate the training of followership prototypes (writing letters of appreciation to followers) into the project of leadership training to enhance leaders’ positive expectations for followers. For example, leaders can generate positive assumption for followers by expressing routinely recalling and focusing on the positive traits and behaviors of their followers (Sy, 2010).

### Limitations and Future Directions

There still exsits some shortcomings in this paper. First, we just investigated the construct of benevolent leadership in Chinese contexts which are characterized by collective culture. However, other countries in Asian, such as Japen and Korea, may share the similar culture with Cheng et al. (2004) found that co-operative (collective) goals can contribute to effective leadership even when leaders and followers own different nationalities (japanese leaders and chinese followers). This phenomenon sheds light on a need to examine the existence of benevolent leadership in other Asian countries.

Second, prior research has found that emotional intelligence is an important determinants of perceived support and career development (Di Fabio and Kenny, 2011, 2012a,b; Di Fabio et al., 2013; Di Fabio and Saklofske, 2014a,b; Di Fabio, 2015; Di Fabio and Kenny, 2015; Di Fabio and Palazzeschi, 2015). Following this logic, leaders with high emotional intelligence may pay more attention to followers’ emotions and show more consideration for their well-being, which are the characteristics of benevolent leadership. Besides, followers with high emotional intelligence may also receive more support from their leaders because they are skilled at communicating with the leaders. Thus, we suggest that future research should investigate the antecedents of benevolent leadership (or supportive leadership) from the emotional intelligence congruence perspective.

Third, the study probes only into the direct effect of implicit–explicit followership congruence on benevolent leadership, whereas the underlying mechanism is not discussed. Lapierre and Bremner (2010) suggested that leaders will trust more in followers when followers’ traits satisfy leaders’ followership prototypes. Hence, the meditating role of trust between followership prototype congruence and benevolent leadership can be a new perspective in future studies. According to the conservation of resources theory, trust in followers induced by PFP–PFT congruence may be a double-edged sword, which may increases followers’ perceived workload and concerns about reputation maintenance in addition to leaders’ consideration for followers (Baer et al., 2015). Thus, we suggest future research should consider both bright and dark sides of PFP–PFT congruence.

Besides, this study explains the relationship between the independent variable and dependent variable in detail, adopting the method of multi-source data to avoid common method variance, but the cross-sectional study design constrains our findings about causality. Thus, future research could take advantage of a longitudinal study design or experimental design to test the impact of implicit–explicit followership congruence on benevolent leadership.

## Conclusion

Drawing on implicit followership theory, the present paper discovered the antecedents of benevolent leadership from the perspective of implicit–explicit followership congruence and demonstrated that recognition-based cognitive processes provide a useful theoretical framework for comprehending leadership phenomena in organizational settings. By matching leaders possessing PFP with followers who possess PFT, organizations can effectively develop benevolent leadership that many scholars deem pivotal for job performance and creativity.

## Author Contributions

JP and XW design the study; JP carried it out, analyzed the results and wrote the manuscript.

## Conflict of Interest Statement

The authors declare that the research was conducted in the absence of any commercial or financial relationships that could be construed as a potential conflict of interest. The reviewer LP and handling Editor declared their shared affiliation, and the handling Editor states that the process nevertheless met the standards of a fair and objective review.

## Footnotes

1 The original questionnaires were first translated by the first author into Chinese versions, which were then back translated by a bilingual linguist we invited. At a following stage, we compared the back translated versions to the original ones until they agreed with the original ones. After the whole process of translation was carried out, we sent the PFP and PFT questionnaires separately to two leader–follower dyads for revision, including over whether the statements were clear and accurate.
